# The Intestinal Expulsion of the Roundworm *Ascaris suum* Is Associated with Eosinophils, Intra-Epithelial T Cells and Decreased Intestinal Transit Time

**DOI:** 10.1371/journal.pntd.0002588

**Published:** 2013-12-05

**Authors:** Dries Masure, Tao Wang, Johnny Vlaminck, Sarah Claerhoudt, Koen Chiers, Wim Van den Broeck, Jimmy Saunders, Jozef Vercruysse, Peter Geldhof

**Affiliations:** 1 Department of Virology, Parasitology and Immunology, Faculty of Veterinary Medicine, Ghent University, Merelbeke, Belgium; 2 Department of Medical Imaging and Small Animal Orthopaedics, Ghent University, Merelbeke, Belgium; 3 Department of Pathology, Bacteriology and Avian Diseases, Faculty of Veterinary Medicine, Ghent University, Merelbeke, Belgium; 4 Department of Morphology, Faculty of Veterinary Medicine, Ghent University, Merelbeke, Belgium; University of Melbourne, Australia

## Abstract

*Ascaris lumbricoides* remains the most common endoparasite in humans, yet there is still very little information available about the immunological principles of protection, especially those directed against larval stages. Due to the natural host-parasite relationship, pigs infected with *A. suum* make an excellent model to study the mechanisms of protection against this nematode. In pigs, a self-cure reaction eliminates most larvae from the small intestine between 14 and 21 days post infection. In this study, we investigated the mucosal immune response leading to the expulsion of *A. suum* and the contribution of the hepato-tracheal migration. Self-cure was independent of previous passage through the liver or lungs, as infection with lung stage larvae did not impair self-cure. When animals were infected with 14-day-old intestinal larvae, the larvae were being driven distally in the small intestine around 7 days post infection but by 18 days post infection they re-inhabited the proximal part of the small intestine, indicating that more developed larvae can counter the expulsion mechanism. Self-cure was consistently associated with eosinophilia and intra-epithelial T cells in the jejunum. Furthermore, we identified increased gut movement as a possible mechanism of self-cure as the small intestinal transit time was markedly decreased at the time of expulsion of the worms. Taken together, these results shed new light on the mechanisms of self-cure that occur during *A. suum* infections.

## Author Summary

*Ascaris lumbricoides* is the most common intestinal parasite in humans. *A. suum* is closely related to *A. lumbricoides* but infects pigs and can be used to study the immune response against larval stages. Most larvae are eliminated from the small intestine between 14 and 21 days after infection in what is called a self-cure reaction. The remaining larvae after this point will be able to grow into adults and reproduce. We show here that the intestinal self-cure of *A. suum* is locally triggered as part of an innate immune defense mechanism. When pigs received lung stage larvae, they were still able to eliminate the parasite, indicating that passage through the liver or lungs is not essential to eliminate the larvae upon their return in the small intestine. We could identify a decrease in the intestinal transit time at 17 days post infection, indicating an increase in gut movement, which could explain why the worms were being driven out at this time.

## Introduction

In (sub)tropical countries, *Ascaris lumbricoides* is an important soil transmitted helminth, infecting around 1 billion people worldwide. Although most cases are sub-clinical, *Ascaris* infections lead to malabsorption and malnutrition and in rare cases obstruction or puncture of the intestinal wall and penetration of the bile and pancreatic ducts occur [1]. The closely related roundworm *A. suum* is one of the most common parasites in pigs causing economic losses in agriculture due to increased feed conversion rate and liver condemnation [2]. Because of the identical life cycle, the high genetic similarity between these parasites [3], and because *A. suum* is a zoonosis [4], [5], *A. suum* infections in pigs make an ideal model for *A. lumbricoides* infections in humans. Cross infections and gene flow between the 2 species also occurs [6], [7], which led to the debate whether or not they belong to the same species [8], [9].

After ingestion, the *A. suum* eggs hatch and release third stage larvae (L3) in the intestine. The larvae will penetrate the caecal or colonic wall, reach the lungs via the liver, after which they will be coughed up and swallowed back in. Once back in the small intestine, they will develop first into L4 and L5 stage larvae and eventually into adults, preferentially inhabiting the proximal half of the small intestine [10]. Immunity against invading third stage larvae takes several weeks of exposure to infective eggs to develop [11], [12]. In contrast, even in primary infections an expulsion mechanism, termed self-cure, causes the elimination of most of the fourth stage larvae (L4) from the small intestine between 14 and 21 DPI, and this self-cure is independent of the inoculation dose [10]. The effector mechanisms driving this elimination are largely unknown.

To date, it is not known if humans infected with *A. lumbricoides* also undergo spontaneous cure. However, in pigs, before self-cure the number of larvae in the small intestine is roughly 30–50% of the infection dose. After 21 DPI, however, the number of larvae is greatly aggregated, with the majority harboring low numbers of worms and a small proportion having the majority of worms [10]. This overdispersion is also seen in humans infected with *A. lumbricoides* [13] and is likely caused by a similar reaction. Although predisposition is a multifactorial phenomenon that includes external factors such as exposure, understanding the mechanism of aggregation might also help to explain the predisposition to high or low worm burdens observed in humans [13].

The aim of this study was to investigate in more detail the gastro-intestinal immune response leading to the elimination of L4 *A. suum* larvae from the small intestine and the contribution of the hepato-tracheal migration to the expulsion of the parasite.

## Materials and Methods

### Ethics statement

All animal experiments were conducted in accordance with the E.U. Animal Welfare Directives and VICH Guidelines for Good Clinical Practice, and ethical approval to conduct the studies were obtained from the Ethical Committee of the Faculty of Veterinary Medicine, Ghent University (EC2011/086, EC2009/145 and EC 2013/51) who have also approved the document.

### Animals and parasites

Helminth naive Rattlerow Seghers hybrid piglets of 10 weeks old were used. The animals were routinely checked for *A. suum* by coprological examination and at the start of the experiment 2 sentinel animals from the same pen were euthanized to confirm absence of larval stages. The animals had access to feed and water *ad libitum*.

*A. suum* eggs were obtained from gravid females collected at the local abattoir from pigs that were being processed as part of the normal work of the abattoir. After incubation in 0,1% KCr_2_ for 2 months, embryonation was confirmed by way of light microscopy.

### Experimental design

#### Experimental infection with *A. suum* eggs

Four groups of 5 pigs were used. The animals of 3 groups were infected via oral intubation with 2000 embryonated *A. suum* eggs each and euthanized 10, 17 and 28 days post infection (DPI), respectively. One group was left uninfected and served as the negative control group.

#### Lung stage larvae transfer experiment

Three donor animals were infected with 200.000 embryonated *A. suum* eggs and euthanized with a captive bolt pistol 9 days post infection. This time was chosen to avoid the chance that larvae would not have developed enough to be ready for the change in environment. The lungs of the animals were collected, minced and the homogenate put on a Baermann funnel incubated at 37°C to collect the lung stage larvae. Preliminary experiments showed that the transfer of larvae resulted in an establishment rate of around 50% 2 days after transfer. Within 2 hours after necropsy of the donor animals, 15 naive pigs were orally infected with 1000 lung stage larvae each, in order to have a similar number of larvae in the small intestine as in the *A. suum* egg infected animals. Five animals were killed each at day 2, 7 and 18 post transfer (DPT) respectively.

#### L4 intestinal stage larvae transfer experiment

Three donor animals were infected with 25.000 embryonated *A. suum* eggs and euthanized 14 days post infection with a captive bolt pistol. The content of the small intestines of the animals were collected and the small intestine was washed with 37°C PBS to collect any remaining larvae. The content of the small intestine and the washing was sieved with a 122 µm sieve and put on a Baermann funnel with PBS at 37°C to collect the intestinal L4 larvae. Within 2 hours after necropsy of the donor animals, the L4 larvae were collected from the Baermann funnel and 15 naive pigs were orally infected with 1000 L4 larvae each. Five animals were killed each at day 2, 7 and 18 DPT respectively.

### Post mortem procedure

All animals were fasted before necropsy and then killed with a captive bolt pistol, exsanguinated and the intestines were removed. Samples for RNA extraction and histological analysis of the jejunum were taken 3 meter caudal of the pylorus. The small intestine was further divided in duodenum, jejunum and ileum. The contents of the 3 parts of the small intestine were collected separately and the intestines were rinsed twice with tap water to collect any remaining larvae. The washing was added to the corresponding content and sieved with a 122 µm sieve. *A. suum* larvae were subsequently counted under a microscope.

### RNA extraction, cDNA synthesis and real time PCR assays

Jejunal tissue was immediately snap frozen in liquid nitrogen and stored at −80 until RNA extraction. RNA extraction was performed using Trizol reagent (Invitrogen), combined with an RNeasy mini kit (Qiagen). A DNase treatment was included to prevent genomic contamination. RNA integrity was assessed using a Biorad Experion with a standard sensitivity chip. cDNA was synthesized with a Biorad cDNA synthesis kit, starting from 1 µg of RNA.

Primers for the real time PCR reactions were designed with the Primer3 software [14] and are listed in Table S1. PCRs were run using Fast SYBR Green Master Mix (Applied Biosystems) on an AB StepOnePlus Real-Time PCR System. Primer specificity was confirmed by observing the melting curve and by sequencing PCR products. Gene expression levels were normalized based on housekeeping genes selected using Genorm [15]. Housekeeping genes tested were: *b2m*, *gapdh, hmbs, rpl4, tbp1* and *ywhaz*. The genes selected for normalization were *hmbs* and *tbp1*. Gene transcription levels are expressed as fold change compared to uninfected controls.

### Histological analysis

Tissue samples were washed in PBS, processed with the Swiss roll technique in order to obtain a large surface for histological examination [16] and fixed in either 10% formaldehyde or Carnoy's fixative for 24 h at room temperature. After fixation, the tissues were dehydrated by passage through a series of graded alcohol dilutions, followed by embedment in paraffin. Tissue samples were cut in 4 µm sections. To assess general histopathological damage and the accumulation of eosinophils, formaldehyde fixed samples were routinely stained with haematoxylin-eosin. The length of the villi and depth of the crypts in the jejunum were measured for 20 villi and their corresponding crypts under a microscope using a calibrated micrometer at 100× magnification. Mucosal eosinophils were counted at 400× magnification on 10 fields corresponding to 0,162 mm^2^. Mast cells were counted on toluidine blue stained slides at 200× magnification using a weibel2 graticule [17]. For immunohistochemistry: formaldehyde fixed, paraffin embedded sections were rehydrated and an antigen retrieval step with citrate buffer was included. Endogenous peroxidase activity was blocked using 1% hydrogen peroxide. Sections were stained with rabbit anti-human CD3 (Dakocytomation A/S) to detect intra-epithelial lymphocytes (IELs) or mouse anti-human MAC387 (Serotec) to stain macrophages. Biotinylated secondary antibodies (Dakocytomation A/S) were added and staining was performed using the peroxidase streptavidine complex (Dakocytomation A/S), diaminobenzidine tetrahydrochloride (DAB, Sigma–Aldrich) and H2O2. Sections were counterstained with haematoxylin. Macrophages were counted at 200× magnification using a weibel2 graticule [17] while IELs were counted for 5 villi randomly and expressed as number of IELs per 100 µm villus epithelium.

### ELISA

The *Ascaris*-specific IgA, IgG, IgE and IgM levels in the serum against the L4 larvae were determined using an indirect in-house ELISA. L4 larvae were collected from the small intestines of animals at 14 DPI. The larvae were ground using a mortar and pestle in liquid nitrogen to a fine powder and subsequently dissolved in PBS to which a 1∶1000 dilution of protease inhibitor cocktail (Sigma-Aldrich) was added. After incubating for 2 hours at 4°C, the extract was centrifuged at 10.000 g for 10 minutes. The supernatant was passed through a 0,22 µm filter and stored at −70°C until use. This extract is being referred to as AsL4.

Plates were coated overnight at 4°C with 100 µl of 5 µg/ml AsL4 in 0,05M sodium bicarbonate buffer (pH 9,6). Serum was added at a concentration of 1/100 and HRP-conjugated goat anti-pig IgM (Thermo Scientific), IgG and IgA (Bethyl laboratories) were used as conjugate at a dilution of 1∶50000, 1∶10000 and 1∶5000, respectively. For the detection of pig IgE antibodies, a pig IgE cross-reacting mouse anti-human IgE antibody (Sigma-Aldrich) at 1∶5000 was used in combination with a HRP-conjugated rabbit anti-mouse IgG at 1: 10000. Finally, O-phenylenediamine 0.1% in citrate buffer (pH 5.0) served as substrate and optical density (OD) was measured at 492 nm. All measurements were performed in duplicate.

### Eosinophil degranulation assay

The purification of circulating eosinophils and the degranulation assay were performed as previously described [18]. Reactive oxygen species production was measured using a chemiluminescence assay with PMA 5 µg/ml as positive control, HBSS with Ca^2+^/Mg^+^ as negative control or 1 mM SIN-1 as a ROS donor. Eosinophils from 1 pig were seeded in a 96-well plate at 2×10^5^ cells/well in 100 µl luminol (1 mM) in HBSS with Ca^2+^/Mg^+^. After 5 min of background measurement at 37°C, 10,20 or 50 *A. suum* L4 larvae collected from infected pigs at 14 DPI were added in 100 µl HBSS, as well as the control agents. To test if there was antibody or complement dependent degranulation, serum from 5 uninfected and 5 animals at 17 DPI was pooled and added at 1/100 dilution. Heat inactivation of serum was done at 58°C for 30 minutes. ROS-production was measured during 120 min in the integration mode. Each condition was performed in triplicate and ROS-production was expressed as the fold change in relative light units (RLU) compared to negative controls (HBSS). The experiment was performed 3 times independent from each other.

### Small intestinal transit time

Eleven pigs were infected with 3000 *A. suum* eggs. Ten days after infection, 2 animals were euthanized to confirm batch infectivity. The small intestinal worm counts in these two pigs were 2019 and 2315. Small intestinal transit time was measured in the remaining 9 pigs at 5 days before infection and at 9, 17 and 35 days after *A. suum* infection. The pigs were starved for 12 hours before barium sulfate was given through gastric intubation at a dosis of 4 ml/kg bodyweight. Lateral and dorso-ventral radiographs were taken every half hour until barium sulfate was located in the colon. If a radiograph was inconclusive about the presence of contrast material in the colon, it was repeated after 10 minutes. The time it took for the barium to reach the colon was recorded as the small intestinal transit time. After the last transit time measurement, the animals were euthanized and worms were collected.

### Statistical analysis

For statistical analysis, GraphPad Prism software (v5.0c) was used. Since a non-Gaussion distribution could be expected, differences between the infected groups and uninfected animals were tested using a nonparametric Kruskal-Wallis test with Dunn's multiple comparison post hoc tests. Finally, a repeated measures Friedman test with Dunn's multiple comparisons post hoc test to find differences in transit time between the different time points.

## Results

### Bypassing the hepato-tracheal migration does not impair the self-cure reaction

Animals were either orally infected with 2000 *A. suum* eggs or with 1000 9-day-old L3 that were collected from the lungs of donor animals. The worm counts are summarized in Table 1. For egg infected pigs, the average total worm count at 10 DPI was 312, with 19% of larvae present in the duodenum, 73% in the jejunum and 9% in the ileum. At 17 DPI, the average total number of larvae present was reduced to 19, most of which were present in the ileum. By 28 DPI, all animals were negative for *A. suum*.

**Table 1:** Worm counts in the small intestine during an infection with 2000 *A. suum* eggs or 1000 lung stage larvae.

		Duodenum	Jejunum	Ileum	Total
Egg infection	10 DPI	58 (64)	227 (82)	27 (48)	312 (90)
	17 DPI	0 (0)	5 (6)	14 (20)	19 (26)
	28 DPI	0 (0)	0 (0)	0 (0)	0 (0)
L3 transfer	2 DPT	1 (1)	384 (35)	26 (23)	411 (35)
	7 DPT	0 (0)	220 (374)	267 (337)	487 (376)
	18 DPT	0 (0)	0 (0)	0 (0)	0 (0)

Numbers shown are the average (SD) of 5 animals.

When animals were orally infected with lung L3's obtained from donor animals, they were still able to eliminate the larvae. At 2 DPT, 38% of transferred larvae were recovered, almost exclusively from the jejunum, indicating a successful transfer. At 7 DPT, although the total number of worms was similar to that of 2 DPT, 50 % of the larvae were now present in the ileum. At 18 DPT, no larvae could be recovered from the animals.

### *A. suum* specific antibodies are not essential in the self-cure response

**Figure 1:**
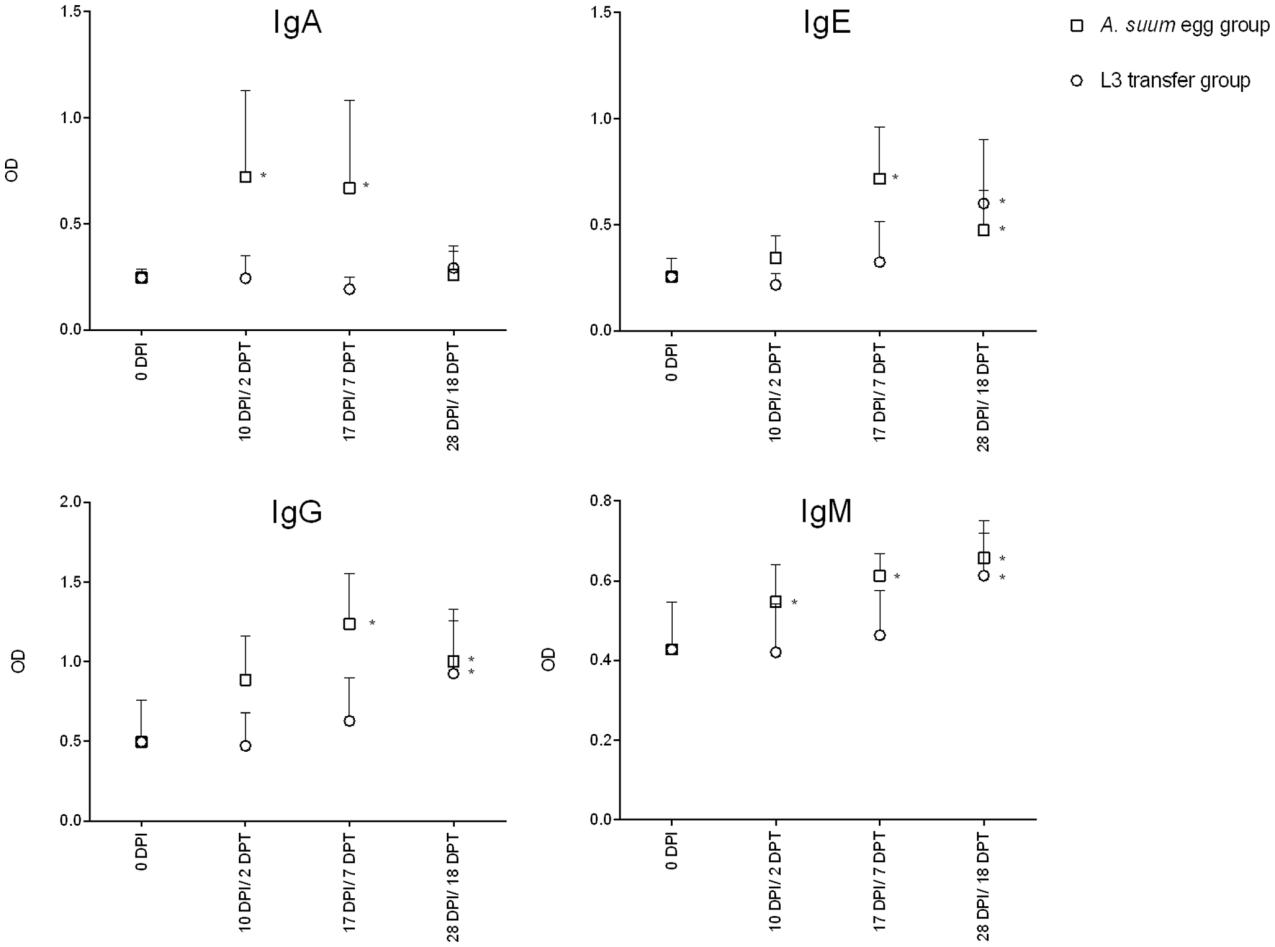
Serum antibodies are present during expulsion in infections with *A. suum* eggs, but not when larvae are transferred. Values represent the mean + SD of 5 animals. * p<0,05 compared to uninfected controls.

*Ascaris* L4 specific IgA, IgE, IgG and IgM antibody levels in serum of *A. suum* egg or lungs stage infected animals were measured using an indirect ELISA (Figure 1). During infections with eggs, AsL4 specific IgA, IgM and IgG levels were increased from 10 DPI onwards, whereas AsL4 specific IgE levels were only detectable in serum at 17 DPI. Although the self-cure reaction occurs 7 days after lung L3's are transferred, no statistically significantly increases of AsL4 specific IgA, IgM, IgG and IgE antibodies could be detected at this time. IgM, IgG and IgE levels were significantly increased only at 18 DPT whereas no change in serum IgA levels was observed.

### L4 transferred larvae are driven distally in the small intestine, but counteract this effect by 18 DPT

We examined whether the release of antigens during the molt from L3 to L4 that occurs around D12 is necessary to trigger the expulsion of the larvae. Therefor we collected 14-day-old L4 intestinal larvae from donor animals and transferred 1000 larvae orally into naïve animals. The number of larvae in each section of the small intestine was counted at 2, 7 and 18 days post transfer. The larvae counts are summarized in Table 2. At 2 DPT around 60% of the transferred larvae could be recovered and 87% of the recovered larvae are present in the jejunum. Five days later the total number of larvae in the small intestine is similar to that at 2 DPT, but most larvae are present in the terminal part of the small intestine. At 18 DPT the total number of larvae has not decreased compared to 7 DPT, but 90% of larvae are now present again in the jejunum, indicating that they could counteract the peristaltic movement to inhabit the proximal region of the small intestine.

**Table 2:** Worm counts in the small intestine after transfer of 1000 *A. suum* L4 larvae.

	Duodenum	Jejunum	Ileum	Total
2 DPT	1 (1)	521 (208)	73 (54)	595 (201)
7 DPT	0,2 (0,4)	102 (148)	317 (262)	419 (369)
18 DPT	24 (53)	409 (175)	19 (43)	452 (199)

Numbers shown are the average (SD) of 5 animals.

### Self-cure is associated with eosinophilia and intra-epithelial T cells

**Figure 2:**
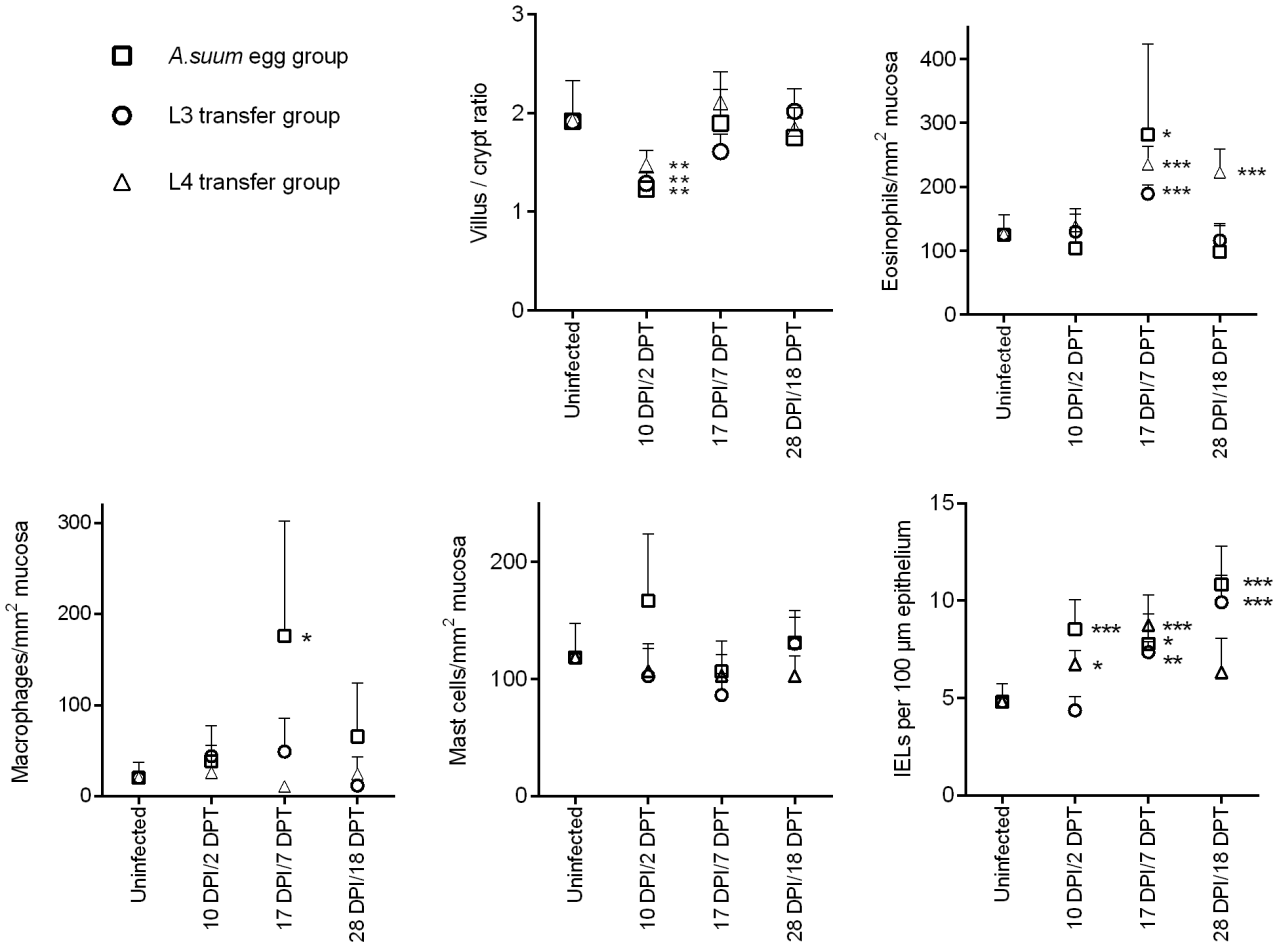
Histopathological findings during infections with *A. suum* eggs and infections with L3 or L4 transferred larvae. Values are mean + SD of 5 animals. * p<0,05 versus control group; ** p<0,01 versus control group; *** p<0,001 versus control group.

The results of the histological parameters investigated are shown in Figure 2. To assess general histopathological changes, villus length and crypt depth were measured. Villus/crypt ratios decreased shortly after contact with *A. suum* larvae, due to a blunting of the villi. Although this effect was observed in both infections with eggs and L3 and L4, it was only temporary, as the villi recovered by 17 DPI/7 DPT. At 17 DPI, coinciding with the expulsion of the parasite, there was a significant increase in mucosal eosinophils. After elimination of the larvae, i.e. 28 DPI, the number of eosinophils decreased to a level similar to that before the infection. A similar pattern was observed following transfer of L3's, with a peak in eosinophil counts at 7 DPT. The transfer of L4 larvae resulted in high eosinophil numbers at 7 DPT and 18 DPT.

Mucosal macrophages followed a similar pattern as eosinophils in *A. suum* egg infected pigs, with a 9-fold increase in the number of macrophages per mm^2^ mucosa at 17 DPI that returned to baseline level at 28 DPI. In contrast to normal infections, in both L3 and L4 transfer infections, no increase in the number of macrophages was observed at any of the time points investigated. No statistically significant changes were observed in the number of intestinal mast cells in any of the infection experiments. Finally, intra-epithelial T cells were significantly elevated in all infection experiments at the time when larvae were being driven towards the distal end of the small intestine, i.e. at 17 DPI/7DPT. In the *A. suum* egg infections and in the L3 transfer experiment, IELs were still elevated even after the worms were eliminated, while in the L4 transfer experiment the IELs returned to normal levels at 18 DPT.

The results of the quantitative PCR analysis on a set of 25 genes for egg infected, L3 and L4 transferred animals are summarized in Table 3. With egg infections, the gene expression pattern was polarized towards a Th1-like response, with significant upregulations observed for *ifng, il12a, il12b, stat4* and *nos2a*. In contrast, none of the Th2 related genes were significantly impacted during infection with *A. suum* eggs. In the L3 and L4 transfer experiments, more mixed responses were measured. In addition to some Th1 markers, an increase in the typical Th2 transcripts *il4* and *il13*, together with increases in regulatory transcripts, such as *foxp3*, and *tgfb* were observed.

**Table 3:** RNA transcription profile of *A. suum* egg infected animals and L3 and L4 infected animals.

Gene	Description	Egg infection			L3 transfer			L4 transfer		
		10 DPI	17 DPI	28 DPI	2 DPT	7 DPT	18 DPT	2 DPT	7 DPT	18 DPT
	**Th1 associated**									
*il12a*	Interleukin 12 subunit p35	2.29**	1.28	1.48	1.15	1.98	1.17	1.45	0.84	0.56
*il12b*	Interleukin 12 subunit p40	2.28*	2.63	2.12*	2.05*	2.11**	1.98	1.26	1.09	1.26
*nos2a*	Nitric oxide synthase 2a, inducible	2.11	5.12*	8.81**	1.19	1.24	0.97	2.41	1.35	1.43
*ifng*	Interferon γ	4.30**	2.91*	3.49*	1.31	2.27	1.02	2.65*	1.44	1.03
*tbx21*	T-Box 21, T-bet	2.28*	1.63	2.05	2.35*	1.59	1.83	1.37	1.04	1.06
*stat4*	Signal transducer and activator of transcription 4	1.66*	0.96	1.70*	1.31	1.15	1.33	6.49*	3.84*	3.42*
	**Th2 associated**									
*il4*	Interleukin 4	1.59	0.76	1.16	1.24*	0.91	0.92	1.04	0.93	1.35
*il5*	Interleukin 5	1.06	0.92	0.84	0.79	0.91	0.80	0.78	0.59*	0.53*
*il13*	Interleukin 13	0.52	1.19	1.32	3.36	1.50*	1.60*	1.38	2.66	40.25**
*stat6*	Signal transducer and activator of transcription 6	0.74	0.77	0.86	0.69	1.39	0.89	0.76	0.74	0.81
*il25*	Interleukin 25	1.36	1.20	1.95	1.27	0.83	0.89	0.41*	0.92	1.59
*il33*	Interleukin 33	1.24	1.07	1.05	0.73	0.50	0.54	0.69	0.71	1.11
*cma1*	Mast cell chymase 1	0.87	0.68	0.94	0.80	0.54*	1.04	0.89	0.68	0.68*
	**Treg**									
*foxp3*	Forkhead box P3	0.89	0.98	1.47	1.55	1.64*	1.95*	1.54	2.10	2.05*
*tgfb*	transforming growth factor β	0.99	0.99	1.15	1.33	1.89*	1.32	1.76**	1.92	1.04
*il10*	Interleukin 10	1.13	1.74	1.33	0.84	1.25	0.59**	1.84	1.65	1.33
*pparg*	peroxisome proliferator-activated receptor gamma	0.42*	0.81	1.35	2.60*	0.83	1.11	0.74	0.89	1.53
	**Cytotoxic cell associated**									
*nkl*	NK-lysin	0.52	1.57	1.16	0.54	0.71	0.92	1.97	1.90	0.97
*gzma*	Granzyme A	1.61**	1.46	2.74**	2.03	2.05*	2.25	1.59*	1.46	0.24**
*gzmb*	Granzyme B	4.14*	2.66	3.69	2.58**	1.32	2.34	3.74**	2.11*	1.04
*prf1*	Perforin 1	1.50	0.83	1.61	1.67*	1.68**	1.70*	2.61*	1.86	1.37
*klrk*	killer cell lectin-like receptor subfamily K, NKG2D	2.71**	1.11	2.28*	1.25	1.02	1.42	0.73	0.41	0.44*
	**Eosinophil associated**									
*epx*	Eosinophil peroxidase	2.64*	0.33	0.41*	0.48	0.44	0.68	0.43	0.73	3.47**
*ccl11*	Chemokine (C-C motif) ligand 11, Eotaxin 1	1.03	0.79	0.98	1.25	2.17*	1.38	0.88	0.79	0.87
*ccr3*	Eotaxin receptor	2.28*	1.32	0.79	0.86	0.83	1.06	0.82	0.88	0.94
*il5ra*	Interleukin 5 Receptor, alpha	1.21	0.59	1.09	1.72	1.19	2.67*	1.42	1.32	1.33

Results are shown as average fold change versus uninfected controls.

* p<0.05 compared to uninfected controls.

** p<0.01 compared to uninfected controls.

For all infection experiments there was an upregulation of genes associated with cytotoxic cells, mainly granzyme A and B, perforin 1 and NKG2D. Additionally, several eosinophil-associated genes were induced, such as those encoding for eosinophil peroxidase, eotaxin 1, eotaxin receptor and IL-5 receptor alpha.

### Eosinophils do not degranulate in response to L4 larvae

**Figure 3:**
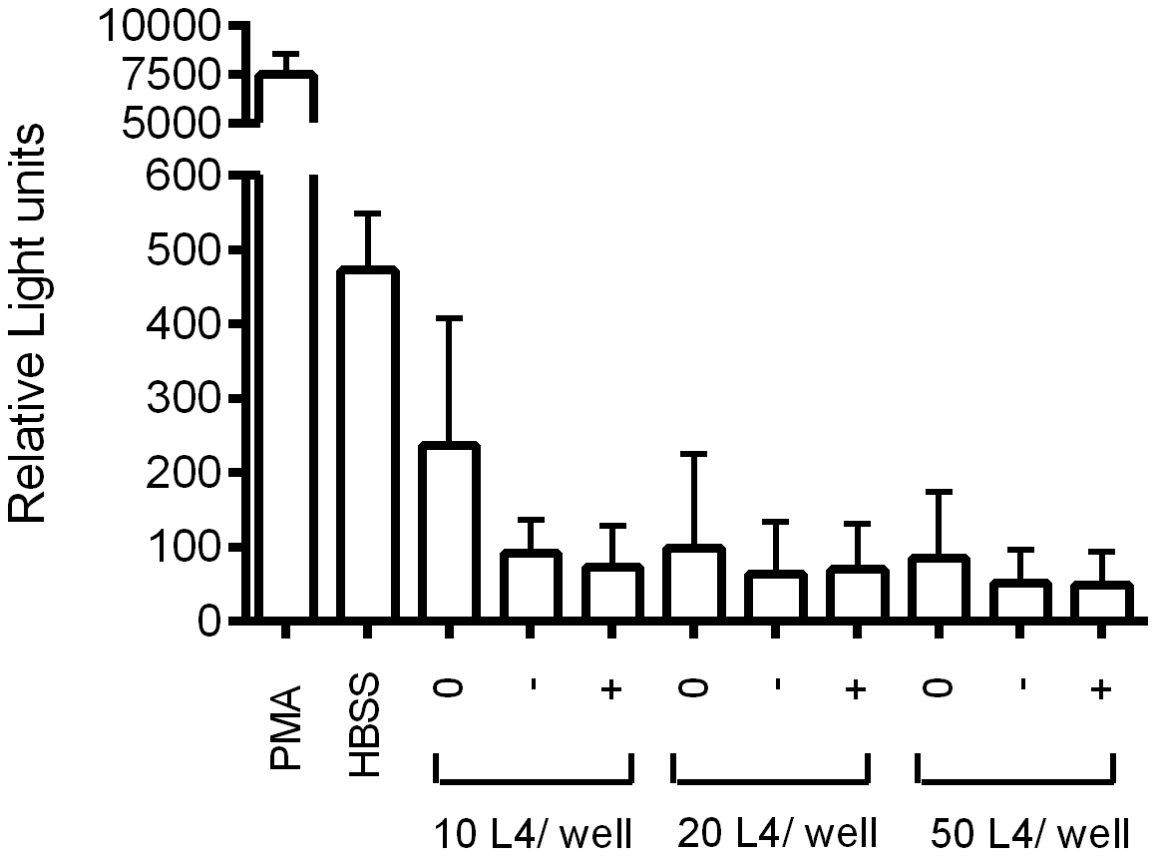
No ROS release by eosinophils in response to *A. suum* L4 larvae. Data are shown as the mean RLU ± SEM of three independent experiments. PMA (5 µg/ml) and HBSS were used as a positive and negative control, respectively. 0: no serum added to the wells; -: serum from uninfected animals added to the wells; +: serum from 17 DPI animals added to the wells. ROS: Reactive oxygen species.

Results of the eosinophil degranulation assay are shown in Figure 3. Measurement of the reactive oxygen species (ROS) indicated that the eosinophils did not degranulate after incubation with *A. suum* L4 larvae, even in the presence of serum from infected animals. To exclude the possibility that L4 larvae would capture ROS released in the medium, *A. suum* L4 larvae were cultured together with SIN-1, a molecule that releases NO and ROS. *A. suum* L4 larvae together with SIN-1 in medium gave no significant differences in measured ROS compared to SIN-1 without L4 larvae (1636±704 RLU versus 977±344 RLU, respectively).

### Small intestinal transit time is decreased during self-cure

**Figure 4:**
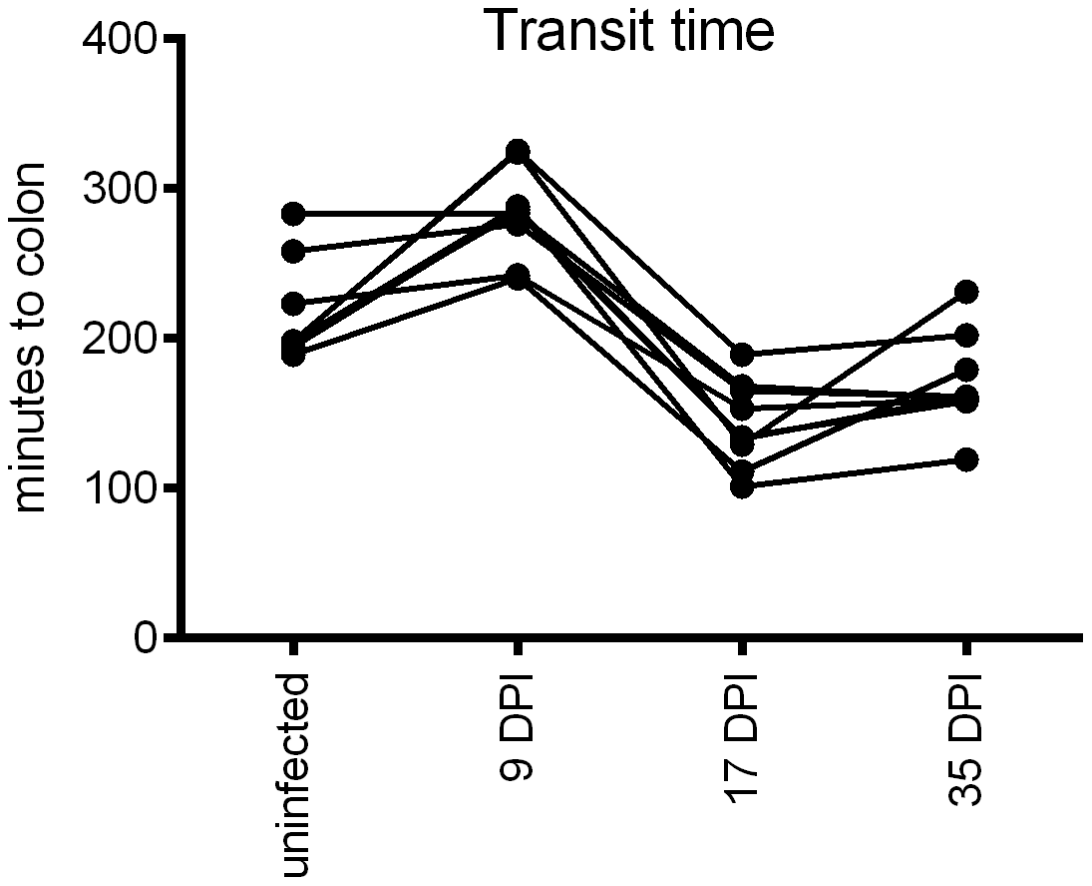
The small intestinal transit time decreases during self-cure. The time for the barium solution to reach the caecum or colon after gastric intubation was recorded in 9 animals before and during *A. suum* infection.

The small intestinal transit time was measured by following barium sulfate passage through the small intestine before infection and at 9, 17 and 31 days post infection with 3000 *A. suum* eggs (see Figure 4). There was a small, non-significant increase in the small intestinal transit time at 9 DPI compared to their pre-infection transit time. At 17 DPI the small intestinal transit time was significantly lower than before the infection. By 35 DPI, 8 out of 9 animals were *A. suum* negative and one pig had 29 *A. suum* worms. At this time, the intestinal transit time was still somewhat lower than before infection, but not significantly.

## Discussion

Here we investigated the immunological basis of the self-cure reaction during primary *A. suum* infections. In addition, we studied the influence of the migration of the larvae through the body on the self-cure reaction. By transferring lung stage *A. suum* larvae from one animal to another, we have a simple model to study the effect of tissue migration on the initiation of the self-cure response. In animals bypassing the passage through the liver and lungs, the self-cure reaction occurred with the same kinetics as animals receiving infectious eggs, i.e. around 7 days after contact with the small intestine. Furthermore, both in infections with eggs and with lung stage larvae all larvae were expelled by 18 days of exposure to the small intestine. A previous study by Jungersen *et al.* led to the speculation that the expulsion of *A. suum* might be affected when the liver is bypassed. They injected *in vitro* hatched L3's intravenously in pigs and found a higher percentage of animals harboring adult *A. suum* at 70 DPI than what is usually observed, even though at 14 DPI there were comparable numbers of L4 larvae between intravenously and orally infected animals [19]. Unfortunately, not enough time points and control groups were included to confirm if previous priming in the liver was indeed required to eliminate the larvae from the small intestine. The results presented here now unequivocally show that the self-cure mechanism is a locally triggered phenomenon, independent of previous passage through the liver or lungs.

Additionally we sought to determine whether antibodies play and important role during self-cure. Since in normal infections there are already *A. suum* specific antibodies present at 10 DPI, it was previously suggested that antibodies played an important role in the expulsion of the parasite [20]. Although we confirm the presence of antibodies during self-cure in egg infected pigs, the absence of *A. suum* specific antibodies when larvae were being expelled in animals that received lung stage larvae would indicate that *A. suum* specific antibodies do not have a major role in the early self-cure against *A. suum*. This is further supported by the observation that when L4 larvae are transferred, most larvae were being driven to the distal end of the small intestine around 7 DPT. However, it still remains possible that both specific and non-specific antibodies present in the mucosa itself could contribute to the *A. suum* expulsion [21]. In addition, although it is not clear to what extent maternal antibodies would still be present in pigs of 12 weeks age, the passive transfer of antibodies has also been shown to contribute to parasite expulsion [22]. Therefore, it would be interesting in future studies to also analyse the mucosal antibody responses.

Remarkably, and in contrast to the transfer of lung stage larvae, the L4 transferred larvae were able to return to the jejunum by 18 DPT. By this time, the larvae are already 32 days old, i.e. an age at which in natural infections they are also not affected by the self-cure response anymore. It appears that these larvae, once they have developed to a certain stage, are able to counteract the self-cure response. This is in agreement with a microarray study on larvae in the jejunum and ileum during self-cure, where they found that only the more metabolically active larvae could remain in the jejunum [23]. However, from our results it is clear that the larvae present in the ileum are still alive and can return to the jejunum if they are active enough. This also contradicts the suggestion that self-cure is a parasite-driven suicide phenomenon based on the density of the parasites [24]. It indicates that there is a fine balance between the host that is trying to drive the parasite out and the parasite's ability to counteract this effort. This also explains why adults can remain in the small intestine for months or years without being driven out.

The histological and RNA transcription analysis showed some common characteristics associated with the expulsion of larvae in all the experiments performed here. The peak of expulsion coincided with a peak in mucosal eosinophils and IELs, suggesting an important role for these cells in the innate defense against *A. suum*. Eosinophils can directly respond to a broad spectrum of pathogens through signaling via Toll like receptors, complement receptors and immunoglobulin receptors. In order to investigate whether eosinophils responded directly to *A. suum* L4 larvae, we monitored the release of reactive oxygen species from the eosinophilic granules after co-incubating the cells with the larvae. In contrast to results obtained with freshly hatched L3's, where eosinophil degranulation occurred quickly after contact with L3's in the presence of serum of either infected or uninfected animals [18], eosinophils did not respond directly to L4 *Ascaris*, even in the presence of serum. In addition, the larvae in the L4 transferred animals at 18 DPT were seemingly unharmed, even though eosinophil numbers remained high. These results may indicate that the L4 larvae are expressing inhibitory factors that prevent eosinophil degranulation and that eosinophils are better equipped to deal with tissue-residing larvae, rather than lumen dwelling ones. This seems indeed the case for many helminth infections [25]. The function of eosinophils in the defense against L4 larvae might also be of an indirect nature. Since the eosinophils were located deep in the mucosa, this assumption seems indeed likely. Through the release of preformed cytokines, chemokines, lipid mediators and cytotoxic molecules, eosinophils could quickly initiate a potent immune response after recognition of pathogen-associated molecular patterns, which in turn may lead to the initiation of the expulsion of *A. suum*.

Another important finding was a clear increase in the number of intra-epithelial T cells during the course of the infection. Although the IELs were not phenotyped, RNA transcription data would suggest that it was the cytotoxic T cell subset that was the most impacted, as there was an overall induction of molecules associated with cytotoxicity such as granzymes, perforin and NKG2D, all of which have been found to be expressed by IELs [26]. One of the functions of IELs is epithelial repair [26]. IELs may be activated in response to damage caused by the larvae. For example, Granzyme B has been found to be correlated with villus damage in helminth infections [27]. Our findings support this, as in all our experiments villous blunting and granzyme B upregulation were observed shortly after contact with *A. suum* larvae. The negative effect of *A. suum* on the intestinal structure might have important consequences for humans suffering from *A. lumbricoides* as well, as it might help to explain the malabsorption often associated with these infections [28]. Whether there is a direct effect of the IELs on the expulsion of the parasite deserves further attention, since resistance against helminth infections in sheep has been associated with genes involved in cytotoxicity [29]. Increased epithelial turnover and shedding caused by cytotoxic cells might make it harder for the small L4's to stick to the mucosa. Interestingly, IELs were lower in the L4 transferred group at 18 DPT, which may indicate an active regulation of the immune response by these larvae.

Mast cells and basophils have previously been associated with *A. suum* infections [18], [30], [31]. Repeated infections induced blood basophilia and intestinal mastocytosis, and these cells responded to stimulation with L3 or L4 secretory antigens by releasing histamine [30], [31]. The maximum histamine release occurred between 14 and 21 days after daily exposure, therefor it has been suggested that these cells played an important role during self-cure [32]. However, only basophils or mast cells that had previously been exposed to *Ascaris* released histamine following contact with L3 or L4 secretory antigens [30], [31]. We also show here that in contrast to experiments with repeated infections, mast cells were not induced in the small intestine after primary infections, suggesting that basophils or mast cells may only play a role in protection against secondary infections.

Interestingly, the local cytokine response in the jejunum seemed to be greatly impacted by the initial migration through the body. Naturally infected animals were more biased towards a Th1 type response with macrophages, while in both the L3 and L4 transfer experiments there was a much more mixed Th1/Th2 response and no recruitment of macrophages. Especially the animals infected with L4 larvae showed high *il13* transcription at 18 DPT, which may indicate that the initial Th1 bias shifts towards a Th2 response as the infection progresses.

Together, these results suggest that the expulsion mechanism does not target the *A. suum* larvae directly. One possible mechanism by which larvae could be eliminated from the small intestine is increased gut movement. We show here that animals infected with *A. suum* indeed have decreased transit time around 17 DPI. This decrease is in agreement with a previous study showing an increase in smooth muscle contractility from 14 to 21 DPI and an increase in fluid secretion *ex vivo* [33]. Any increase in gut movement would indeed make it more difficult for the relatively small larvae to remain in the small intestine and may in fact be a universal mechanism of expulsion of intestinal lumen dwelling nematodes, as changes in intestinal smooth muscle contractility have been identified in *Cooperia oncophora* infected calves and *Trichinella spiralis* and *N. brasiliensis* infected mice [34]–[36]. Studies in mice have shown that the helminth induced increase in smooth muscle contractility is signaled through IL4 or IL-13 [36]–[38], which could explain why it is a common observation with helminth infections. Of particular interest is the contribution of alternatively activated macrophages on the regulation of smooth muscle contractility [36]. While we could only detect an increase in macrophages in the *A. suum* egg infected animals, it remains possible that changes in the activation state of macrophages contribute to the change in smooth muscle contractility.

Taken together, this study indicates that the self-cure is a locally initiated mechanism., Faster gut movement will make it harder for the larvae to remain in the small intestine. It is also part of a weep and sweep response that is often associated with helminth infections and which consists of increased luminal secretion (weep) and increased gut movement (sweep) [39]. This effect can probably be overcome once *A. suum* larvae have developed to a point where they are large and active enough to counteract the increased peristaltic movements. Eosinophils and intra-epithelial T cells appear to play a pivotal role since they are consistently associated with self-cure, but further research is needed to elucidate how these cells operate in order to induce the weep and sweep response.

## Supporting Information

Table S1 **Primer sequences.**
(DOC)
