# Supplementary material for: The Intestinal Expulsion of the Roundworm Ascaris suum Is Associated with Eosinophils, Intra-Epithelial T Cells and Decreased Intestinal Transit Time
Source: PLoS Negl Trop Dis. 2013 Dec 5;7(12):e2588. doi: 10.1371/journal.pntd.0002588 (PMC3854935; doi:10.1371/journal.pntd.0002588)
Supplement: Table S1 — Primer sequences. (DOC) [file pntd.0002588.s001.doc]

**Table S**1: Primer sequences

| **Gene** | **Forward primer** | **Reverse primer** | **Accession number** |
| --- | --- | --- | --- |
| *b2m* | CACTCCTAACGCTGTGGATCAG | CCACTTAACTATCTTGGGCTTATCG | AB436775.1 |
| *ccl11* | CTTCTGTCGCCACCATCTG | ATTCTCTTGGGCATCAGCAC | XM_003131725.1 |
| *ccr3* | ACAATGTTCTGCATCTGACCTAAAAT | AGAATGGAAAGAACCAGCTCTGTCT | NM_001001620 |
| *epx* | TGGCCTCCCAGGGTACAAT | CAGGAACTTCCTCGCCAAAG | Ssc.33169 |
| *foxp3* | GGTGCAGTCTCTGGAACAAC | GGTGCCAGTGGCTACAATAC | AY669812 |
| *gapdh* | GGCATGGCCTTCCGTGT | GCCCAGGATGCCCTTGAG | DQ845173.1 |
| *gzma* | GGAGCTCACTCGATAACCAAGAAA | GCTTTAGAAGTTTAAGGTCACCCTCAT | NM_001198926.1 |
| *gzmb* | TCTCCTATGGAAGAAAGGATGGAA | ATCCAGGGCAGGAAACTTGA | NM_001143710 |
| *hmbs* | GCACGGCCATGTCTGGTAAC | CCACCACACTGTCCGTTTGTAT | NM_001097412 |
| *ifng* | TGGTAGCTCTGGGAAACTGAATG | GGCTTTGCGCTGGATCTG | AY188090 |
| *il10* | TGAGAACAGCTGCATCCACTTC | TCTGGTCCTTCGTTTGAAAGAAA | NM_214041 |
| *il12a* | GGCCTGCTTACCACTTGAAC | GCATTCATGGCCTGGAACTC | NM_213993 |
| *il12b* | CTGAAGAAGACGGCATCACG | AGGAGTGACTGGCTCAGAAC | NM_214013 |
| *il13* | CTGACCACCAGCATGCAGTACT | GCTGCAGTCGGAGATGTTGA | NM_213803 |
| *il25* | GAACCCACACCTTCCATTTG | ATCTCCAGAGGAGGCATGAG | XM_001926286.2 |
| *il33* | AGCTTCGCTCTGGCCTTATC | GCTGACAGGCAGCAAGTACC | XM_003121912.1 |
| *il4* | GCCGGGCCTCGACTGT | TCCGCTCAGGAGGCTCTTC | NM_214123 |
| *il5* | TGGTGGCAGAGACCTTGACA | CCATCGCCTATCAGCAGAGTT | AJ010088 |
| *il5ra* | CAAGGATGCCCCTGAGGA | TGCTGTATTCTTGGCATTCTTCA | XM_003358500.2 |
| *klrk1* | TCTCAAAATTCCAGTCTTCTGAAGATATA | AGGATCTGTTTGTTGGAATTTGTACTA | NM_213813 |
| *nkl* | GTCTGACCCCTGAGCACTCT | CCCAGCTCCTCTCTTTGGAG | XM_003124939.1 |
| *nos2a* | CGTTATGCCACCAACAATGG | AGACCCGGAAGTCGTGCTT | NM_001143690 |
| *rpl4* | CAAGAGTAACTACAACCTTC | GAACTCTACGATGAATCTTC | DQ845176.1 |
| *stat4* | ACCATTCGCTGACATCCTTC | TGGGAGCTGTAGTGTTTACC | XM_001924928.1 |
| *stat6* | TCCCAGCTACGATCAAGATG | AGTGAGAGTGTGGTGGATAC | HM135386.1 |
| *tbp1* | AACAGTTCAGTAGTTATGAGCCAGA | AGATGTTCTCAAACGCTTCG | DQ178129 |
| *tgfb* | GAAGCGCATCGAGGCCATTC | GGCTCCGGTTCGACACTTTC | NM_214015 |
| *ywhaz* | ATGCAACCAACACATCCTATC | GCATTATTAGCGTGCTGTCTT | XM_001927228.2 |
